# Blood microRNA Signatures Serve as Potential Diagnostic Biomarkers for Hepatic Sinusoidal Obstruction Syndrome Caused by *Gynura japonica* Containing Pyrrolizidine Alkaloids

**DOI:** 10.3389/fphar.2021.627126

**Published:** 2021-02-19

**Authors:** Xunjiang Wang, Wei Zhang, Yongfeng Yang, Yiran Chen, Yuzheng Zhuge, Aizhen Xiong, Li Yang, Zhengtao Wang

**Affiliations:** ^1^ The MOE Key Laboratory for Standardization of Chinese Medicines and the SATCM Key Laboratory for New Resources and Quality Evaluation of Chinese Medicines, Institute of Chinese Materia Medica, Shanghai University of Traditional Chinese Medicine, Shanghai, China; ^2^ Shanghai R&D Center for Standardization of Traditional Chinese Medicines, Shanghai, China; ^3^ Department of Gastroenterology, The Drum Tower Hospital of Nanjing, Affiliated to Nanjing University Medical School, Nanjing, China; ^4^ Department of Liver Disease, The Second Hospital of Nanjing, Affiliated to Nanjing University of Chinese Medicine, Nanjing, China

**Keywords:** Gynura japonica, pyrrolizidine alkaloids, hepatic sinusoidal obstruction syndrome, microRNA, diagnosis, biomarker

## Abstract

**Background and Aims:** The *Gynura japonica*-induced hepatic sinusoidal obstruction syndrome (HSOS) is closely related to pyrrolizidine alkaloids (PAs), and its prevalence has been increasing worldwide in recent years. However, no effective therapy for PA-induced HSOS in clinics is available, partially due to the failure of quick diagnosis. This study aims to identify blood microRNA (miRNA) signatures as potential biomarkers for PA-induced HSOS in clinics.

**Methods:** The microarray-based miRNA profiling was performed on blood samples of the discovery cohort, which consisted of nine patients with HSOS and nine healthy donors. Differentially expressed miRNAs were further confirmed using a validation cohort, which consisted of 20 independent patients with HSOS. In addition, the rat model was established through the oral administration of the total alkaloid extract from *G. japonica* to investigate the association of miRNA biomarkers with the progression of HSOS. Bioinformatic analyses, including GO and KEGG enrichment, receiver operating characteristics curve, and correlation analyses were conducted to evaluate the accuracy of the potential miRNA biomarkers.

**Results:** Three miRNAs, namely miR-148a-3p, miR-362-5p, and miR-194-5p, were overexpressed in patients and rats with PA-induced HSOS. These miRNAs were positively related to the severity of liver injury and displayed considerable diagnostic accuracy for patients with HSOS with areas under the curve over 0.87.

**Conclusion:** In summary, this study demonstrated that three miRNAs, hsa-miR-148a-3p, hsa-miR-362-5p, and hsa-miR-194-5p, might serve as potential biomarkers for PA-induced HSOS in clinics.

## Introduction

Given its effectiveness and safety, herbal medicine is practiced worldwide, especially in developing countries such as China, Afghanistan, and Ethiopia. However, herbal drug-induced liver injury (HILI) has been increasingly reported worldwide (Shen et al., 2019). The well-known hepatic sinusoidal obstruction syndrome (HSOS) is caused by pyrrolizidine alkaloids (PAs) in *Gynura japonica* (Thunb.) Juel. (syn. *Gynura segetum* (Lour.) Merr., i.e., *Tusanqi* in Chinese), a medicinal herb used for pain relief, hypertension, and dissipation of blood stasis (Chen and Huo, 2010; Kan et al., 2016; Guo et al., 2019; Zhuge et al., 2019).

HSOS is a rare hepatic vascular disease with clinical symptoms of hepatomegaly, ascites, jaundice, and hyperbilirubinemia abdominal distension. Diagnosing PA-induced HSOS is rather difficult. The pathological confirmation by liver biopsy is regarded as the golden criteria for inspecting HSOS. However, patients with PA-induced HSOS usually show extensive ascites, coagulation disorders, and thrombocytopenia (Yang et al., 2019), which makes it difficult to perform a liver biopsy. The Roussel Uclaf Causality Assessment Method is popular for the diagnosis of HILI but is not specific enough for PA-induced HSOS (Zhuge et al., 2019). Pyrrole–protein adducts (PPAs) may serve as potential diagnostic markers for PA-induced HSOS (Xia et al., 2016). However, the contents of PPAs vary remarkably significantly with the amount and the duration of PA intake and sampling time and may be undetected in some patients with PA-induced HSOS. Therefore, a noninvasive and feasible biomarker is needed for the early and accurate diagnosis of PA-induced HSOS in clinical practice.

Several microRNAs (miRNAs) have demonstrated potential to become novel noninvasive biomarkers for detecting liver toxicity (Yamaura et al., 2012; Krauskopf et al., 2015; Teschke et al., 2016; Howell et al., 2018). miRNAs are a class of small (about 22 nucleotides) endogenous noncoding RNAs that negatively regulate gene expression at the post-transcriptional level by binding to the 3′-untranslated region of target mRNAs, resulting in mRNA degradation or translational repression (Ameres and Zamore, 2013). When secreted into the circulation, miRNAs exhibit aberrant expression under different physiological and pathological conditions and exist stably (Wang et al., 2009). Therefore, the expression of miRNAs contributes to the progression and outcomes of several types of disease. Recently, a few reports have evaluated the changes in miRNAs in liver injury by monocrotaline (MCT) (Huang et al., 2017; Oda et al., 2018; Takeuchi et al., 2018), a toxic PA compound. For example, Huang et al. (2017) found that 11 miRNAs are significantly altered in the liver of mice treated with MCT, indicating that miRNAs are involved in PA-induced liver injury. However, only laboratory work has been performed. Moreover, the MCT-induced hypertension in rodents (Schultze and Roth, 1998) is not observed in patients with PA-induced HSOS. The presence of such a pathology caused by MCT is likely to have a significant physiological effect, which may also affect the miRNA profiling.

Therefore, a retrospective study is performed in this study to map the miRNA profile in PA-induced HSOS and uncover the miRNA signatures for potential diagnostic biomarkers for PA-induced HSOS.

## Materials and Methods

### Study Design, Patients, and Healthy Donors

Study subjects were recruited from Drum Tower Hospital, the Affiliated Hospital of Nanjing University School of Medicine (Jiangsu Province, China). A total of 32 patients admitted to the hospital between February 2017 and February 2019 were included in this retrospective study. All patients were diagnosed with HSOS in accordance with the previously described criteria (Yang et al., 2019) and with the chief complaint of self-medication of *G. japonica*. Three patients with other known hepatic etiologies, i.e., autoimmune, alcoholic, and nonalcoholic liver diseases, were excluded. The enrolled 29 patients with PA-induced HSOS were randomly grouped into two cohorts, namely, cohort 1 (*n* = 9) for the discovery phase and cohort 2 (*n* = 20) for the validation phase. Age- and sex-matched healthy donors (*n* = 9) were recruited from subjects who participated in routine physical examinations at the same hospital. Signed informed consent was obtained from all participants prior to enrollment, allowing the analysis of blood samples and all clinical data. The Ethics Committee of Nanjing Drum Tower Hospital approved the study. This study was designed and performed in accordance with the ethical guidelines of the 1975 Helsinki Declaration.

### Whole Blood Collection and RNA Extraction

A total of 2.5 ml fasting venous blood was collected from patients with PA-induced HSOS or healthy volunteers and stabilized in the PAXgene Blood RNA Tubes (PreAnalytiX, Hombrechtikon, Switzerland). The whole blood was allowed to stand at room temperature for 2 h and stored at −80 °C until subsequent analysis. Total RNA >18 nucleotides (including miRNA) were extracted and purified using the PAXgene Blood miRNA Kit (PreAnalytiX, Hombrechtikon, Switzerland) in accordance with the manufacturer’s instructions. The RNA Integrity Number of the extracted RNA was checked to inspect the RNA integration by using the Agilent Bioanalyzer 2,100 (Agilent technologies, Santa Clara, CA, United States). The RNA purity and concentration were determined using the Nanodrop ND-1000 spectrophotometer (Nanodrop Technologies, Wilmington, DE).

### miRNA Profiling and Data Analysis

Agilent Human miRNA (8*60K) V21.0 (i.e., human microRNA microarrays from Agilent Technologies, which contains probes for 2,549 human microRNAs from the miRbase database v.21.0) were used in this study. The miRNA molecules in the total RNA were labeled using the miRNA Complete Labeling and Hyb Kit (Agilent technologies, Santa Clara, United States). Each slide was hybridized with 1.65 μg Cy3-labeled cRNA by using the Gene Expression Hybridization Kit (Agilent technologies, Santa Clara, United Sstates) in accordance with the manufacturer’s instructions. The hybridized arrays were washed, fixed, and scanned using the Agilent Microarray Scanner (Agilent technologies, Santa Clara, United States). The Agilent Feature Extraction software (version 10.7) was used to analyze the acquired array images. Raw data were normalized using the Quantile algorithm and the limma packages in the R software (version 3.5.1; MathSoft, Seattle, WA). Differentially expressed miRNAs between healthy donors and patients with PA-induced HSOS were screened using the criterion of false discovery rate (FDR) adjusted *p* value <0.05 and fold change (FC) ≥ 2.

### Real-Time Quantitative PCR (RT-qPCR)

For the detection of miRNA expression levels, an equal amount of total RNA (300 ng) was reverse transcribed to generate the cDNA by using the miScript II RT Kit (Qiagen, Venio, The Netherlands) in accordance with the manufacturer’s instructions. qPCR was performed using the miScript SYBR® Green PCR Kit (Qiagen, Hilden, German) in accordance with the manufacturer’s instructions. The conditions for the qPCR were in accordance with the kit protocol. The U6 small nuclear RNA (Qiagen, Hilden, German) was used as the internal control. Specific primers for mature miRNAs were designed using the miR primer algorithm and described in Supplementary Table S1 (Busk, 2014).

### Prediction and Bioinformatics Analysis of miRNA Target Genes

Several computational microRNA-target prediction tools were developed to predict the relationships between miRNAs and their target mRNAs. Three miRNAs, namely, hsa-miR-148a-3p, hsa-miR-362-5p, and hsa-miR-194-5p, were subjected to the TargetScan 7.2 (available at: http://www.targetscan.org/vert_72/) and the miRDB v6 (available at: http://mirdb.org/miRDB/). The intersection of the two databases were estimated using the Venny 2.1 software (http://bioinfogp.cnb.csic.es/tools/venny/). The transcripts, which were identified by both programs, were selected as the potential targets for further analysis. The Gene Ontology (GO) and Kyoto Encyclopedia of Genes and Genomes (KEGG) pathway enrichment analyses were performed using the clusterProfiler package in the R software to identify the functional categories enriched and pathways for the network nodes. The *q* value denoted the significance of GO or KEGG term enrichment in DE genes. A low *q* value indicated a significant GO or KEGG term. In addition, the intersection from the above results was derived, and the genes associated with the biological process of angiogenesis were emphasized.

### Animal Experiments

*G. japonica* was collected from Yangzhou City (Jiangsu Province, China) and authenticated by authors. A voucher specimen was deposited and available in the specimen room of Shanghai R&D Center for Standardization of Traditional Chinese Medicines (No. JG010; Shanghai, China). The total alkaloid extract (TA) was previously prepared (Xiong et al., 2019). TA was subjected to the UPLC–diode-array detection–mass spectrometry (MS) analysis. The contents of PAs in TA were determined in accordance with our previously reported method (Xiong et al., 2019) to ensure the repeatability of the extract. TA was dissolved in acidified 0.9% sodium chloride (pH 6.5) to prepare a solution containing 12 mg TA per ml.

Male 6-week-old Sprague-Dawley rats were obtained from the Laboratory Animal Center of Shanghai University of Traditional Chinese Medicine (SHUTCM, Shanghai, China). All animals were housed in a controlled environment (temperature = 23 °C ± 2 °C, relative humidity = 55 ± 5%, room air changes = 12–18 times/h, and 12 h light on/off cycle), with ad libitum access to food and water. The animal welfare and experimental protocols were strictly compliant with the Guide for the Care and Use of Laboratory Animals and the protocol-related ethics regulations of SHUTCM (Registration number: PZSHUTCM190912019). Rats were randomly divided into four groups with five rats per group. Rats were made to fast for 12 h before being orally dosed with 120 mg/kg TA by single administration and sacrificed at different times after the TA treatment, i.e., 1 h (0.04 days), 3 h (0.13 days), 12 h (0.5 days), and 1, 2, and 7 days. The rats in the control group were treated with the same volume of physiological saline. Rats were made to fast for 12 h and anesthetized with isoflurane to collect blood samples from the descending aorta. Liver tissues were also separated.

### Serum Clinical Biomarker Assay and Histopathological Assessment

After collection, an aliquot of the blood sample (2 ml) was immediately transferred to the PAXgene Blood RNA Tubes for the extraction and purification of total RNA. Others were allowed to stand at room temperature for 30 min to collect serum samples by centrifugation at 1,500 g and 4 °C for 15 min. The serum activities of alanine aminotransferase (ALT) and aspartate aminotransferase (AST) and the levels of three key factors for coagulation function, i.e., prothrombin time (PT), thrombin time (TT), and plasma fibrinogen (FIB), were also examined.

Rat livers were fixed in 4% polyformaldehyde for 24 h, embedded in paraffin, and subsequently sectioned (5 μm) for the hematoxylin and eosin staining. Slices were histologically assessed using light microscopy (Olympus Cor., Tokyo, Japan).

### Quantification of PPAs

The PPAs in sera were determined using a precolumn derivatization liquid chromatography (LC)–tandem MS in accordance with our previous report (Chen et al., 2020). An aliquot of the serum (20 μl) was mixed with 200 μl acetone, vortexed, and centrifuged at 900 g for 5 min. The pellet was washed twice with absolute ethanol and constituted into 200 μl AgNO_3_ ethanol solution (2%) and shaken for 30 min. The mixture was centrifuged at 15,000 g and 4 °C for 5 min. The resulting supernatant was removed and reacted with 800 μl Ehrlich reagent, which contained 134.2 mM DABA in absolute ethanol and 2% (*v/v*) boron trifluoride diethyl ether at 55 °C for 10 min. The sample solution was filtered and subjected to LC–MS on the SHIMADZU CBM-30A-UPLC system (Shimadzu Co., Kyoto, Japan) linked to the ABSCIEX QTRAP6500 (AB SCIEX Co., CA, United States) system in the positive ion mode.

### Statistical Analysis

The statistical analyses of clinical characteristics were performed using the SPSS statistical software version 24.0 (SPSS, Chicago, Illinois, United States). All continuous variables were expressed as medians (25th–75th percentiles) and compared with the Mann–Whitney–Wilcoxon test in accordance with distribution characteristics. Categorical variables were expressed as numbers with percentages and compared using the *χ* ^2^ test or the Fisher’s exact test.

For the qPCR analysis, raw data were normalized against the reference miRNAs that were included in all plates. Relative levels were quantified using the 2^−∆∆^CT method, where ∆Ct = Ct _(target gene)_ − Ct _(reference gene)_. All data after the logarithm transition were subjected to statistical analysis. qPCR statistical analyses were performed in the GraphPad Prism v7 (GraphPad Software, San Diego, United States), and *p* values <0.05 were considered statistically significant. The R software (version 3.5.1; MathSoft, Seattle, WA) was used to run volcano plots, principal component analysis (PCA), correlation analysis, and receiver operator characteristic (ROC) analysis.

## Results

### Characteristics of Study Subjects

**FIGURE 1:**
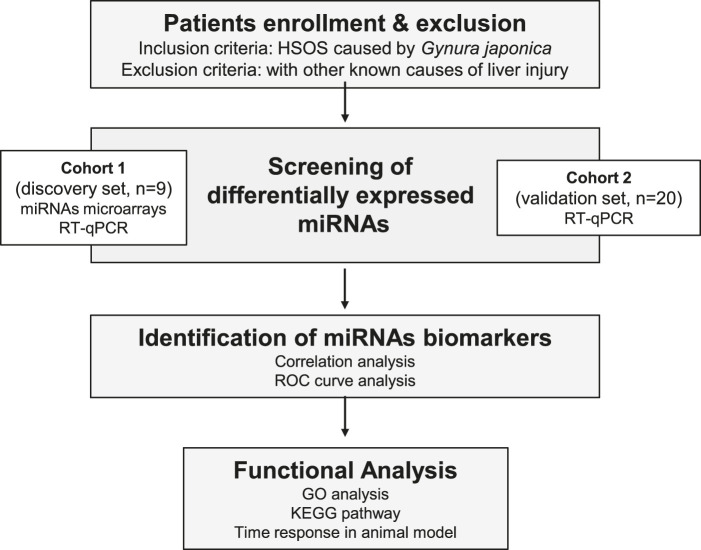
Flowchart of the experimental design.

The flow chart of the experiment for the detection of potential PA-induced HSOS biomarkers is shown in Figure 1. One cohort, including nine healthy controls and nine patients with HSOS, was used as the discovery set, whereas another cohort, including 20 patients with HSOS, was used as the validation set. A detailed description of the clinical characteristics for these participants is described in Table 1. No significant difference in clinicopathological features was observed between the discovery and the validation phases. The primary clinical manifestations of PA-induced HSOS were consistent with the literature (Zhuge et al., 2019). PPAs were detected in 89.7% (26 out of 29) patients with HSOS, with concentration ranging from 0.89 nmol/L serum to 169.31 nmol/L serum.

**TABLE 1:** Clinicopathological characteristics of PA-HSOS patients enrolled in the study.

Characteristics	Biomarker discovery phase			Biomarker validation phase	
	Healthy donors (*n* = 9)	HSOS (*n* = 9)	*p* value	HSOS (*n* = 20)	*p* value
	*n* (%)	*n* (%)	a	*n* (%)	b
Gender^a^(male/female)					
Male *n* (%)	5 (55.56)	6 (66.67)	0.629	17 (85.00)	0.158
Female *n* (%)	4 (44.44)	3 (33.33)		3 (15.00)	
Age^b^(years)	63.00 (63.00, 68.00)	65.00 (64.00, 68.00)	0.387	67.00 (61.50, 67.50)	0.710
Blood coagulation					
PT^b^(s)	12.60 (12.28, 12.90)	18.00 (16.35, 22.85)	0.001	14.90 (14.35, 17.15)	0.007
INR^b^	0.94 (0.91, 0.97)	1.59 (1.42, 2.03)	<0.001	1.31 (1.25, 1.51)	<0.001
APTT^b^(s)	35.60 (34.08, 39.63)	43.40 (37.95, 50.00)	0.029	35.50 (30.20, 37.95)	0.395
TT^b^(s)	17.15 (16.25, 17.85)	19.70 (17.60, 23.40)	0.121	20.10 (19.15, 21.40)	0.003
FIB^b^(g/L)	3.85 (3.75, 4.10)	1.50 (1.31, 2.32)	<0.001	2.00 (1.80, 2.15)	<0.001
D-dimer^b^(μg/mL)	0.39 (0.27, 0.46)	2.50 (1.71, 3.89)	0.029	1.44 (1.04, 2.34)	0.001
Blood routine test					
WBC^b^(10^9^/L)	6.40 (5.90, 7.60)	6.57 (5.75, 9.23)	0.776	5.50 (4.80, 7.50)	0.503
RBC^b^(10^12^/L)	4.17 (3.61, 4.44)	4.84 (4.33, 5.27)	0.050	5.16 (4.58, 5.29)	0.007
PLT^b^(10^9^/L)	180.00 (174.00, 201.00)	120.50 (92.25, 146.50)	0.026	106.00 (76.00, 135.00)	0.016
HGB^b^(g/L)	128.00 (112.00, 141.00)	144.50 (125.75, 178.25)	0.328	154.00 (138.50, 158.00)	0.038
HCT^b^	38.10 (33.90, 39.60)	40.75 (35.13, 45.03)	0.456	46.40 (40.10, 47.40)	0.043
MCV^b^(fL)	92.10 (90.80, 94.30)	84.70 (79.33, 88.05)	<0.001	91.40 (85.90, 93.75)	0.230
RDW-CV^b^(%)	13.20 (12.80, 13.50)	18.45 (14.73, 21.43)	0.001	13.40 (13.10, 14.20)	0.295
Biochemical test					
ALT^b^(U/L)	17.00 (13.00, 38.00)	48.95 (27.93, 158.00)	0.021	76.50 (48.05, 245.30)	<0.001
AST^b^(U/L)	17.00 (15.00, 27.00)	91.60 (40.00, 236.00)	<0.001	73.30 (48.30, 198.50)	<0.001
ALP^b^(U/L)	78.00 (72.00, 96.00)	120.60 (99.68, 142.00)	0.027	108.40 (85.35, 130.00)	0.020
GGT^b^(U/L)	16.96 (14.00, 20.97)	73.80 (53.83, 91.88)	<0.001	85.60 (64.75, 130.40)	<0.001
TBIL^b^(μmol/L)	10.80 (9.62, 13.76)	43.20 (26.68, 122.20)	<0.001	36.50 (26.80, 41.60)	<0.001
DBIL^b^(μmol/L)	1.58 (0.00, 2.30)	31.80 (18.23, 82.53)	<0.001	14.20 (13.05, 22.05)	<0.001
CHE^b^(KU/L)	8.63 (7.52, 9.67)	4.03 (3.39, 4.64)	0.019	2.90 (2.43, 3.40)	0.001
TP^b^(g/L)	68.60 (64.84, 71.78)	52.60 (49.98, 56.70)	0.008	58.50 (55.00, 64.25)	0.046
ALB^b^(g/L)	40.90 (36.93, 42.26)	31.10 (30.23, 35.35)	0.036	35.20 (32.90, 36.30)	0.012
GLO^b^(g/L)	29.60 (25.30, 30.80)	20.95 (19.25, 22.53)	0.015	22.50 (19.85, 28.65)	0.243
A/G^b^	1.40 (1.32, 1.60)	1.65 (1.48, 2.00)	0.236	1.38 (1.17, 1.69)	0.710
TBA^b^(μmol/L)	4.45 (4.13, 4.73)	55.50 (33.30, 85.85)	0.001	42.10 (31.40, 78.15)	<0.001
UREA^b^(mmol/L)	7.31 (4.74, 7.83)	6.40 (6.10, 7.58)	0.743	6.85 (4.33, 8.43)	0.897
CREA^b^(μmol/L)	78.30 (69.86, 98.77)	80.00 (73.00, 92.40)	0.963	88.50 (61.75, 115.75)	0.897
UA^b^(μmol/L)	429.50 (328.53, 468.50)	336.00 (196.80, 365.00)	0.167	417.00 (272.50, 465.75)	0.762
CRP^b^(mg/L)	0.10 (0.05, 0.33)	27.45 (18.33, 32.53)	0.024	23.20 (11.20, 25.40)	0.001

^a^ Chi-square test.

^b^ Median (25th and 75th quartile), Mann-Whitney-Wilcoxon test or Kruskal-Wallis test.

a: Healthy donors VS discovery phase PA-HSOS.

b: Healthy donors VS validation phase PA-HSOS.

A/G, albumin/globulin; ALB, albumin; ALP, alkaline phosphatase; ALT, alanine aminotransferase; APTT, active partial thromboplastin time; AST, aspartate aminotransferase; CHE, cholinesterase; CREA, creatinine; CRP, C-reactive protein; DBIL, direct bilirubin; FIB, plasma fibrinogen; GGT, γ-glutamyl transferase; GLO, globulin; HCT, hematocrit; HGB, hemoglobin; INR, international normalized ratio; MCV, mean corpuscular volume; PA-HSOS, hepatic sinusoidal obstruction syndrome induced by *Gynura japonica* containing PAs; PLT, platelet count; PT, prothrombin time; RBC, red blood cell count; RDW-CV, red blood cell volume distribution width-coefficient of variation; TBA, total bile acid; TBIL, total bilirubin; TP, total protein; TT, thrombin time; UA, uric acid; WBC, white blood cell count.

### Screening of Differentially Expressed miRNAs in Patients With PA-Induced HSOS

**FIGURE 2:**
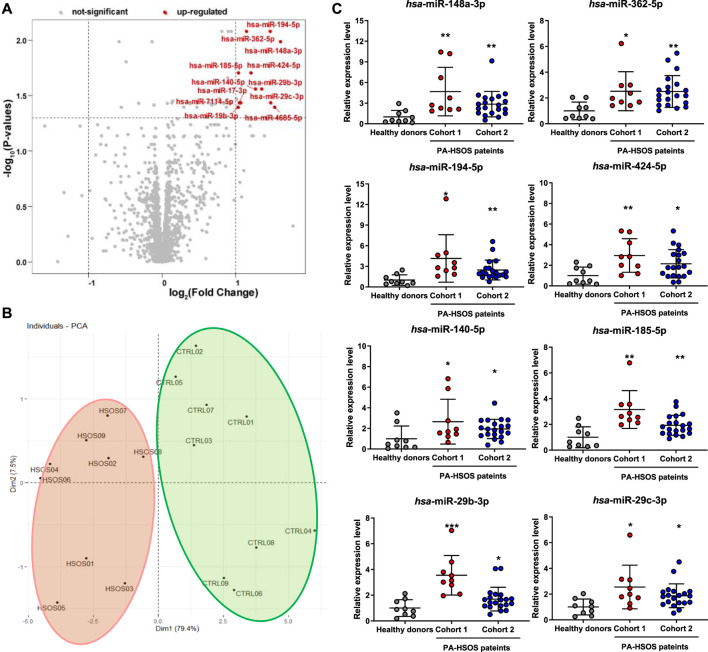
Screening of the differentially expressed miRNAs in patients with PA-induced HSOS. **(A)**, Volcano plots of miRNAs by microarray analysis. The plots were constructed by plotting -log_10_ (FDR adjusted-*p* value) on the *y*-axis and log_2_ (Fold Change) on the *x*-axis. Red blots represent up-regulated differentially expressed miRNAs and gray blots represent miRNAs with no significant difference. **(B)**, PCA score plot of unique differentially expressed miRNAs. **(C)**, Levels of eight differentially expressed miRNAs in patients with PA-induced HSOS. Data were analyzed by Student’s *t* test and expressed as mean ± SD (*n* = 9 in healthy donors, *n* = 9 in cohort 1, and *n* = 20 in cohort 2). *p < 0.05, **p < 0.01, ***p < 0.001 vs. healthy donors.

Blood samples from nine healthy control donors and nine patients with PA-induced HSOS were first subjected to the Agilent Human miRNA (8*60K) V21.0 microarrays to discover differentially expressed miRNAs. The signals of all samples were normalized and evenly distributed (Supplementary Figure S1). Considering the criteria of FC > 2 and FDR adjusted-*P* value <0.05, 12 miRNAs were screened and upregulated in patients with PA-induced HSOS compared with those in healthy donors (Figure 2A; Supplementary Figure S2). The PCA, which was universally used for achieving the natural inter-relationship within the data without prior knowledge of the data set, was used as an unsupervized method to study the differences between healthy donors and patients with HSOS. The score plot obtained using the 12 miRNAs showed clear separation between healthy donors and patients with HSOS (Figure 2B). Ten principal components were calculated using the cross validation, and 86.9% of the variables could be explained by the first two components, indicating a significant difference between healthy donors and patients with HSOS in the profiles and the levels of miRNAs.

The RT-qPCR was performed to further verify the reproducibility of the results from microarray (Supplementary Table S2). Eight miRNAs, namely, hsa-miR-148a-3p, hsa-miR-362-5p, hsa-miR-194-5p, hsa-miR-424-5p, hsa-miR-140-5p, hsa-miR-185-5p, hsa-miR-29c-3p, and hsa-miR-29b-3p, were evidently upregulated in patients with PA-induced HSOS (Figure 2C). Eight candidate miRNAs were further validated by another cohort, i.e., a validation cohort that included 20 patients with PA-induced HSOS (Figure 2C). Similar results were obtained. No significant difference was found between cohorts 1 and 2.

### Identification of Potential miRNA Biomarkers Associated With PA-Induced HSOS

**FIGURE 3:**
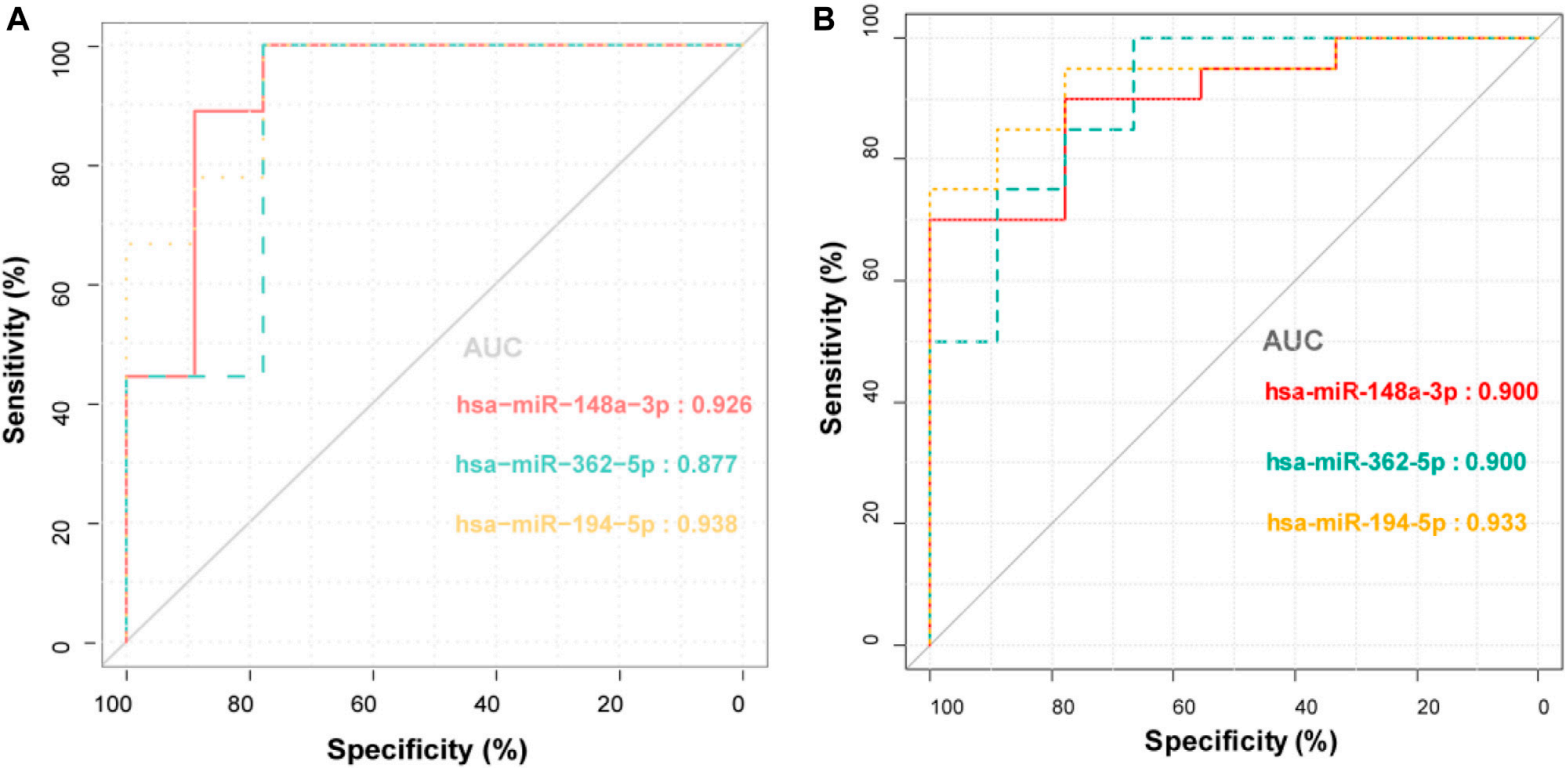
ROC curve analysis for the discrimination between healthy donors and patients with PA-induced HSOS by the three miRNA biomarkers.

The correlation analysis was performed between the levels of differentially expressed miRNAs and clinical indices. The expression levels of three miRNAs, i.e., hsa-miR-148a-3p, hsa-miR-362-5p, and hsa-miR-194-5p, were positively correlated with liver function indices and coagulation parameters, including ALT, AST, alkaline phosphatase, λ-glutamyl transferase, total bilirubin, direct bilirubin, international normalized ratio (INR), TT, and D-Dimer (Table 2). They were also negatively correlated with FIB. Notably, PPAs were only weakly linked to liver function indices. The ROC curve analysis was also carried out to assess the performance of these selected miRNAs to distinguish healthy donors from patients with PA-induced HSOS. All three miRNAs showed good diagnostic accuracy, i.e., area under the curve over 0.87 and Youden index over 0.67 (Figure 3; Supplementary Table S3).

**TABLE 2:** Correlation analysis between the levels of three miRNA biomarkers and PPAs with related indexes in clinical laboratory.

Correlation coefficient	*hsa*-miR-148a-3p	*hsa*-miR-362-5p	*hsa*-miR-194-5p	PPAs
Liver function indexes				
ALT	0.768**	0.689**	0.626**	0.609*
AST	0.775**	0.801**	0.688**	0.529*
ALP	0.453*	0.586**	0.465*	0.074
GGT	0.646**	0.615**	0.674**	0.276
TBIL	0.429*	0.662**	0.518**	−0.219
DBIL	0.416*	0.684**	0.564**	−0.212
Coagulation parameters				
PT	0.474*	0.520**	0.171	0.222
INR	0.686**	0.734**	0.503*	0.337
TT	0.508*	0.596**	0.555**	0.072
FIB	−0.587**	−0.657**	−0.526**	−0.101
D-dimer	0.804**	0.709**	0.501*	0.288

ALT, alanine aminotransferase; AST, aspartate aminotransferase; ALP, alkaline phosphatase; DBIL, direct bilirubin; FIB, plasma fibrinogen; GGT, λ-glutamyl transferase; INR, international normalized ratio; PPAs, pyrrole-protein adducts; PT, prothrombin time; TBIL, total bilirubin; TT, thrombin time. The correlation analyses were performed by the Spearman correlation method. *p < 0.05, **p < 0.01, ***p < 0.001.

### Functional Analysis of PA-Induced HSOS-Associated miRNA Target Genes

**FIGURE 4:**
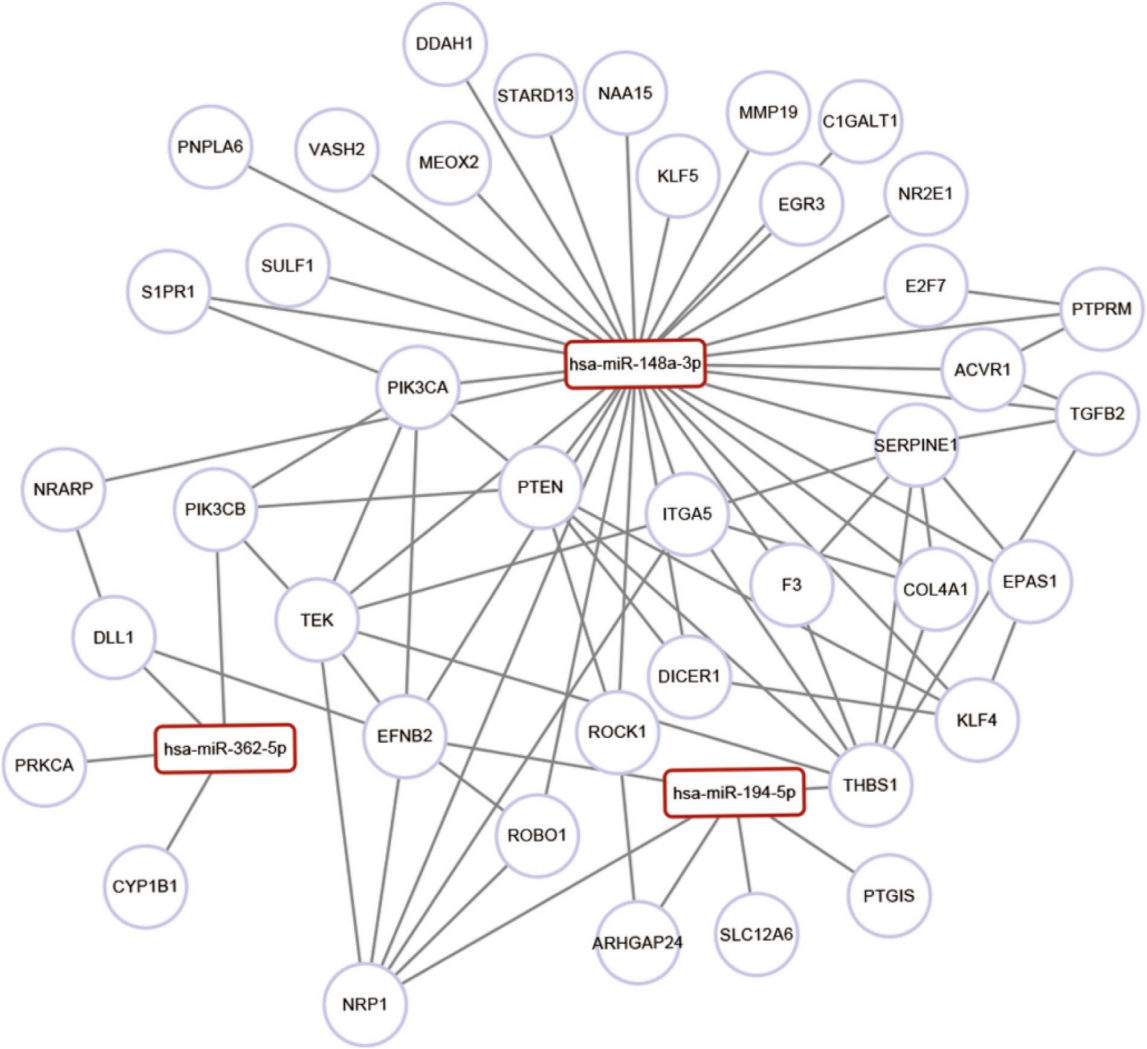
Gene enrichment and pathway analysis of miRNAs target genes. Co-expression networks of angiogenesis-associated genes and co-regulated miRNAs.

The GO and the KEGG analyses of the target genes of the three miRNAs biomarkers, i.e., miR-148a-3p, miR-362-5p, and miR-194-5p, were performed. The top 30 significant GO items were associated with sprouting angiogenesis, cell migration involved in sprouting angiogenesis, cell adhesion, angiogenesis, epithelial tube formation, hepatocyte differentiation, and regulation of fibroblast growth factor receptor signaling pathway (Supplementary Table S4). The KEGG analysis highlighted the importance of the transforming growth factor (TGF)-beta signaling pathway, focal adhesion, extracellular matrix (ECM)–receptor interaction, and regulation of the actin cytoskeleton (Supplementary Table S5). Considering that the HSOS was characterized with angiogenesis-related pathological processes (Zhuge et al., 2019), the co-expression of angiogenesis-associated genes with HSOS-associated miRNAs was investigated. Results indicated that miRNAs might correlate with multiple angiogenic genes (Figure 4; Supplementary Table S6).

### Association of miRNA Biomarkers With the Progression of PA-Induced HSOS

The TA of *G. japonica* was administered to rats to investigate the relationship between the expression of differential miRNA biomarkers and the progression of PA-induced HSOS. Four PAs, i.e., senecionine (264.1 mg/g), seneciphylline (561.7 mg/g), senecionine *N*-oxide (8.7 mg/g), and seneciphylline *N*-oxide (9.1 mg/g), were found with high concentration in TA (Supplementary Figure S3). The total PA content was 843.7 mg/g TA.

**FIGURE 5:**
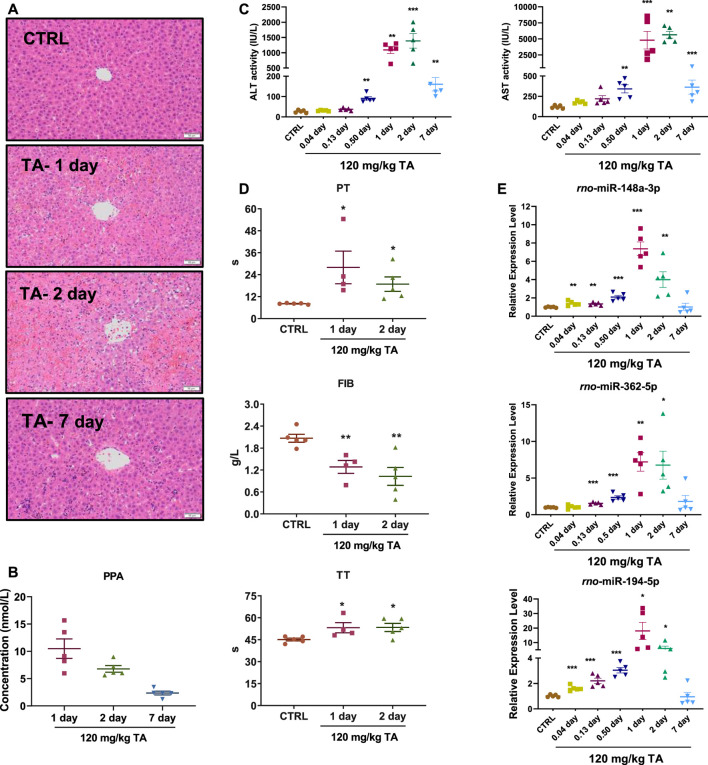
Time-dependent analysis in liver injury and plasma coagulation index levels in rats. Sprague-Dawley rats were orally administered TA at a dose of 120 mg/kg. The rats were euthanized at 0.04, 0.13, 0.5, 1, 2, and 7 days after the administration, and the blood was then collected for analysis. **(A)**, Representative image of hematoxylin and eosin staining of livers (10×). Scale bar, 50 μm. **(B)**, PPAs contents in serum. **(C)**, Serum ALT and AST activities. **(D)**, PT, FIB, and TT levels. **(E)**, Expression levels of miR-148a-3p, miR-362-5p, and miR-194-5p. Data were analyzed by one-way ANOVA and expressed as mean ± SEM. *p < 0.05, **p < 0.01, ***p < 0.001 vs. CTRL group.

Rats were orally administered with TA (120 mg/kg, equivalent to 100 mg/kg PAs), and samples were collected at different time points 1 week after treatment. The histopathological changes in the livers, including scattered foci of hepatocellular necrosis, hemorrhage, and congestion, peaked on day 2 (Figure 5A). The serum ALT and AST activities and PPA contents rapidly increased on days 1 and 2 after treatment and decreased on day 7 (Figures 5B,C). Coagulation function indices, including PT and TT, increased, whereas the FIB level decreased (Figure 5D). The expression of three miRNA biomarkers were evaluated (Figure 5E), and their profiles were highly concordant with those of serum biochemistry and blood coagulation parameters, including ALT, AST, PT, FIB, and TT (Table 3). All results were consistent with those in patients with PA-induced HSOS. Interestingly, two miRNA biomarkers, miR-148a-3p and miR-194-5p, were positively correlated with serum PPA contents (Table 3).

**TABLE 3:** Correlations between three miRNA biomarkers and PPAs with liver function indexes and coagulation parameters in rats treated with TA of *G. japonica.*

Correlation coefficient	rno-miR-148a-3p	rno-miR-362-5p	rno-miR-194-5p	PPAs
Liver function indexes				
ALT	0.623**	0.704**	0.726**	0.729**
AST	0.652**	0.673**	0.718**	0.729**
Coagulation parameters				
PT	0.846**	0.839**	0.854**	0.500
FIB	−0.717**	−0.797**	−0.738**	0.250
TT	0.746**	0.646*	0.746**	0.333
Other				
PPAs	0.796*	0.475	0.893*	—

Rats were orally treated with TA of G. japonica and sacrificed at 1, 2 and 7 days after treatment. ALT, alanine aminotransferase; AST, aspartate aminotransferase; FIB, plasma fibrinogen; PPAs, pyrrole-protein adducts; PT, prothrombin time; TA, total alkaloids extract of G. japonica; TT, thrombin time. The correlation analyses were performed by the Spearman correlation method. *p < 0.05, **p < 0.01, ***p < 0.001.

## Discussion

In this study, we found that *G. japonica* containing PAs caused severe HSOS, which was characterized by abnormal liver function and coagulation systems in humans and rodents. Three miRNAs, namely, hsa-miR-148a-3p, hsa-miR-362-5p, and hsa-miR-194-5p, were screened and validated to be the potential biomarkers for PA-induced HSOS in clinics by further analyses, including qRT-PCR, correlation analysis, and ROC curve analysis.

miRNAs are detectable in many biofluids, such as blood, urine, and feces. miRNAs are promising biomarkers especially when measured from blood or other easily accessed body fluids. Therefore, miRNAs have potential as minimally invasive disease markers (Liu et al., 2018). Several miRNAs are regarded as the noninvasive biomarkers of drug-induced liver injuries (DILI) (Hornby et al., 2014). For example, the miR-122 is upregulated in the capillary blood obtained using the finger venipuncture in patients with DILI (Vliegenthart et al., 2017). Oda et al. (2018) have reported that the miR-511-3p in plasma is a possible biomarker for the liver sinusoidal endothelial cell (LSEC) damage characterized by miRNA expression in LSECs and hepatocytes. A few laboratory works were also performed using rodents to uncover the potential miRNA biomarkers for PA-induced HSOS. Takeuchi et al. (2018) have suggested that the LSEC-derived miR-21-5p and miR-511-3p in serum serve as early-phase biomarkers in response to the LSEC damage and that hepatocyte-enriched miR-122-5p, miR-192-5p, and miR-101b-3p serve as indicators for the hepatocyte damage in MCT-induced HSOS in rodents. In the present study, two independent cohorts were used to uncover the miRNA signatures related to PA-induced HSOS in clinics for the first time. Three miRNAs (i.e., hsa-miR-184a-3p, hsa-miR-194-5p, and hsa-miR-362-5p) had great potential to serve as biomarkers for PA-induced HSOS in humans and rodents after further validation. The expression levels of miR-122-5p and miR-192-5p, which were reported to be dramatically increased in rodents treated with chemical hepatotoxins (such as thioacetamide, acetaminophen, and MCT), were verified. However, large variations were observed among different patients with PA-induced HSOS (Supplementary Figure S4), suggesting that miR-122-5p and miR-192-5p might not be stable enough to serve as biomarkers.

Chen and Duan (2020) have reported that miR-148a-3p is upregulated in the peripheral blood of patients after hepatectomy and positively correlated with serum ALT and AST activities, suggesting that the miR-148a-3p may be sensitive to ischemic and traumatic liver injury. Elevated levels of blood miR-148a-3p is also reported in patients with chronic hepatitis B (CHB), cirrhosis, and hepatocellular carcinoma (Jin et al., 2019). miR-194-5p and the miR-184a-3p are potential biomarkers for CHB infection (Van Der Ree et al., 2017). A single miRNA is frequently not specific to the disease, whereas miRNA signatures that consist of a plurality of different miRNAs may help improve the differentiation between pathologies (Backes et al., 2016). The clinical manifestations of PA-induced HSOS show dramatic changes in liver function and the blood coagulation. In the present study, hsa-miR-184a-3p, hsa-miR-194-5p, and hsa-miR-362-5p were found to be closely related to the occurrence and the development of PA-induced HSOS and positively related to the severity of liver injury and the abnormal fibrinolytic system. For example, an extremely high correlation was found between the hsa-miR-148a-3p and the D-Dimer and indicated hyperfibrinolysis in patients with PA-induced HSOS. As a global indicator of coagulation activation and fibrinolysis, the D-Dimer serves as an indirect marker of thrombotic activity (Bates, 2012), and an increased D-Dimer level is correlated with several hepatic and vascular pathogeneses (Zhang et al., 2010; Saray et al., 2012). During early wound healing, platelets are activated and recruited to the wound site where clot formation and fibrinolysis occur. The FIB is crosslinked by the transglutaminase factor XIIIa and converted into fibrin to form fibrin-rich clots. Subsequently, the fibrinolytic system degrades fibrin to dissolve the clot, whereas the D-Dimer is cleaved from fibrin (Danese et al., 2007). Negative correlations are also found between miRNA signatures and FIB in patients with PA-induced HSOS.

The function analysis of PA-induced HSOS-associated miRNA-targeted genes showed that these PA-induced HSOS-associated miRNAs were co-expressed with genes enriched in angiogenesis, ECM–receptor interaction, focal adhesion, and TGF-beta signaling pathway. Liver fibrosis plays an important role in the pathogenesis of PA-induced HSOS and is closely related to the TGF-beta/p-Smad3 signaling pathway and the activation of proinflammation factors (Lu et al., 2019; Zhang et al., 2019). Thrombospondin-1 (TSP-1) is among the most important activators of TGF-beta signaling and can further lead to liver dysfunction (Jefferson et al., 2020). The TSP-1 inhibitory peptide inhibits the TGF-beta signal activation in mice with 70% hepatectomy and accelerates cell proliferation after surgery (Kuroki et al., 2015). Consistently, genes targeted by HSOS-associated miRNAs are associated with the repression of the antiangiogenic factor TSP-1 in our study. Hepatic fibrosis is closely related to the activation and the proliferation of hepatic stellate cells, the excessive secretion of collagen protein, and the deposition of ECM. The present study indicated that miRNAs were involved in the regulation of vascular endothelial cell injury and coagulation, which was consistent with the previous report (Zhuge et al., 2019). The vascular endothelial growth factor can stimulate the proliferation of sinusoidal endothelial cells and hepatocytes during liver regeneration (Taniguchi et al., 2001; Bockhorn et al., 2007). The endothelial injury caused by PAs triggers the coagulation cascade and induces a hypercoagulable state (Chen and Huo, 2010). The elevation of the plasminogen activation inhibitor-1 is regarded as a characteristic marker of HSOS. This hypothesis was also proven in the present study. Plasmin, which is converted from the circulating zymogen plasminogen, is a potent thrombolytic protease that can dissolve fibrin blood clots and degrade ECM directly or indirectly by activating matrix metalloproteinases (Davis et al., 2001; Sternlicht and Werb, 2001; Law et al., 2013). The cell adhesion is an integrated process involved with cytoskeletal dynamics and cellular tension. The adhesion formation and disassembly affect the migration cycle and the cytoskeletal polymerization. The adhesion assembly is also involved in signaling regulation (Parsons et al., 2010). The vascular cell adhesion molecule 1 (VCAM1) can mediate the leukocyte endothelial cell adhesion. A previous report (Akil et al., 2015) has suggested the important role of VCAM1 in the development of HSOS. Overall, the three miRNA signatures may modulate several key pathways involved in liver function and blood coagulation in PA-induced HSOS.

Most PAs are metabolically activated by cytochrome P450 enzymes to form dehydropyrrolizidine alkaloids, which are active intermediates and highly electrophilic to rapidly interact with cellular macromolecules, thereby forming adducts (such as PPAs) to initiate the toxic effect. Thus, PPAs are suggested as potential diagnostic markers for PA-induced HSOS (Xia et al., 2016; Zhuge et al., 2019). However, as the xenobiotic metabolites of PAs, the PPA contents in serum are greatly affected by several key factors. In the present study, 29 patients with PA-induced HSOS were confirmed by clinical examination. The PPA was not detected in three patients, whereas varying PPA contents, ranging from 0.89 nmol/L serum to 169.31 nmol/L serum, were observed in other patients. PPA contents were found to be highly correlated with serum ALT and AST activities in patients and rodents with PA-induced HSOS, but no relevance was found between the PPA content and coagulation parameters, which were also the key clinical manifestations of PA-induced HSOS in clinics. The expression levels of three miRNA biomarkers in blood were highly correlated with several coagulation parameters, including PT, INR, TT, FIB, and D-Dimer (Table 2). Interestingly, the miR-184a-3p and the miR-362-5p levels were positively related to the serum PPA contents in rats with PA-induced HSOS (Table 3) but not in patients with PA-induced HSOS. An important finding was that the levels of miR-148a-3p, miR194-5p, and miR-362-5p increased in rats as early as 3 h (i.e., 0.13 day) after TA treatment whereas the conventional serum clinical biochemical indicators ALT and AST increased at 12 h after TA treatment (Figures 5C,E). Therefore, the three miRNA signatures were more stable than PPAs and might be good biomarkers for PA-induced HSOS in clinics.

Several limitations might be involved in the present study. First, the high sensitivity and specificity of the biomarkers identified in the present study made them useful for real-time clinical testing and early clinical intervention. However, only limited numbers of patients were enrolled in our study because of the rarity of this clinical presentation. The small sample size might lead to selection bias. However, results from the microarray were validated using a dependent cohort containing 20 patients with HSOS, and the correlation between the levels of differentially expressed miRNAs and conventional clinical indices was analyzed. Furthermore, a time response study was performed in rats, which suggested similar results as those in patients with HSOS. These investigations may help enhance the strength of using these miRNA markers as promising prognostic biomarkers in clinics. Therefore, our ongoing project might further confirm results by performing large multiple-center prospective studies to establish clinically useful cutoffs for their future use in clinical trials. Second, the enrichment analyses of altered miRNA abundance were predicted using bioinformatics tools in the present study. Further investigation should be performed to elucidate the detailed molecular mechanisms of candidate miRNAs alone or in combination in the pathogenesis of PA-induced HSOS.

## Conclusion

In summary, the miRNA profiling after the PA exposure was performed in clinical samples for the first time. Three blood miRNAs (namely, hsa-miR-148a-3p, hsa-miR-362-5p, and hsa-miR-194-5p) were suggested as new promising biomarkers for PA-induced HSOS in clinics. Our results provided further information on the diagnosis of HSOS induced by herbal medicines and preparations containing PAs.

## Data Availability

The datasets presented in this study can be found in online repositories. The names of the repository/repositories and accession number(s) can be found below: https://www.ncbi.nlm.nih.gov/geo/GSE164635.

## Abbreviations

ALP, alkaline phosphate; APTT, activated partial thromboplastin time; AST, aspartate aminotransferase; AUC, areas under the curve; DBIL, direct bilirubin; FC, fold change; FDR, false discovery rate; FIB, fibrinogen; GGT, γ-glutamyl transpeptidase; GO, Gene Ontology; HSOS, hepatic sinusoidal obstruction syndrome; INR, international normalized ratio; KEGG, Kyoto Encyclopedia of Genes and Genomes; PAs, pyrrolizidine alkaloids; PPAs, pyrrole-protein adducts; PT, prothrombin time; ROC, receiver operating characteristics; RT-qPCR, real-time quantitative polymerase chain reaction; SD, standard deviation; TA, total alkaloids; TBIL, total bilirubin; TT, thrombin time.
