# Supplementary material for: Blood microRNA Signatures Serve as Potential Diagnostic Biomarkers for Hepatic Sinusoidal Obstruction Syndrome Caused by Gynura japonica Containing Pyrrolizidine Alkaloids
Source: Front Pharmacol. 2021 Feb 19;12:627126. doi: 10.3389/fphar.2021.627126 (PMC7933570; doi:10.3389/fphar.2021.627126)

Supplemental tables

Supplemental Table 1. Sequences of the primers used for RT-qPCR.

Supplemental Table 2. Validation of the 8 differentially expressed miRNAs in healthy donors and PA-HSOS patients by RT-qPCR.

Supplemental Table 3. ROC curve analysis for the discrimination between healthy donors and PA-HSOS patients by the three miRNA biomarkers.

Supplemental Table 4. Top 30 GO annotations for genes targeted by the differentially expressed miRNAs.

Supplemental Table 5. Top 30 KEGG pathways for genes targeted by the differentially expressed miRNAs.

Supplementary Table 6. The intersection between 3 potential miRNAs biomarkers and angiogenesis pathway genes.

Supplementary Table 1. Sequences of the primers used for RT-qPCR.

Systematic Name	Sequence (5'-3')	Accession number
hsa-miR-140-5p	CAGCAGTGGTTTTACCCTATG	MIMAT0000431
hsa-miR-148a-3p	GCAGTCAGTGCACTACAGA	MIMAT0000243
hsa-miR-17-3p	CTGCAGTGAAGGCACTT	MIMAT0000071
hsa-miR-185-5p	CAGTGGAGAGAAAGGCAGT	MIMAT0000455
hsa-miR-194-5p	CAGTGTAACAGCAACTCCA	MIMAT0000460
hsa-miR-19b-3p	AGTGTGCAAATCCATGCAA	MIMAT0000074
hsa-miR-29b-3p	CAGTAGCACCATTTGAAATCAG	MIMAT0000100
hsa-miR-29c-3p	CAGTAGCACCATTTGAAATCG	MIMAT0000681
hsa-miR-362-5p	CGCAGAATCCTTGGAACCT	MIMAT0000705
hsa-miR-3653-3p	CGCAGCTAAGAAGTTGAC	MIMAT0018073
hsa-miR-4685-5p	CCCAGGGCTTGGAGTG	MIMAT0019771
hsa-miR-7114-5p	GTCTGTGGAGTGGGGT	MIMAT0028125

Supplemental Table 2. Validation of the 8 differentially expressed miRNAs in healthy donors and PA-HSOS patients by RT-qPCR.

Systematic Name	HSOS patients in cohort 1 (n=9)			HSOS patients in cohort 2 (n=20)		
	Fold change	*p* value	*p* value summary	Fold change	*p* value	*p* value summary
hsa-miR-148a-3p	4.7	0.0080	**	2.9	0.0089	**
hsa-miR-194-5p	4.2	0.0168	*	2.5	0.0082	**
hsa-miR-29b-3p	3.6	0.0003	***	1.7	0.0131	*
hsa-miR-185-5p	3.2	0.0014	**	1.9	0.0067	**
hsa-miR-424-5p	3.0	0.0057	**	1.9	0.0293	*
hsa-miR-140-5p	2.7	0.0244	*	1.9	0.0299	*
hsa-miR-29c-3p	2.6	0.0199	*	1.9	0.0165	*
hsa-miR-362-5p	2.5	0.0141	*	2.5	0.0020	**

Data were analyzed by Student's t test and expressed as mean±SD. *$p<0.05$, **$p<0.01$, ***$p<0.001$ versus healthy donors.

Supplemental Table 3. ROC curve analysis for the discrimination between healthy donors and PA-HSOS patients by the three miRNA biomarkers.

Index	HSOS patients in cohort 1 (n=9)			HSOS patients in cohort 2 (n=20)		
	hsa-miR-148a-3p	*hsa*-miR-362-5p	*hsa*-miR-194-5p	*hsa*-miR-148a-3p	*hsa*-miR-362-5p	*hsa*-miR-194-5p
AUC	0.926	0.877	0.938	0.900	0.900	0.933
(95 % Cl)	(0.80; 1.00)	(0.70; 1.00)	(0.83; 1.00)	(0.80; 1.00)	(0.75; 1.00)	(0.82;1.00)
Sensitivity %	100.00	100.00	100.00	70.00	100.00	75.00
Specificity %	77.78	77.78	77.78	100.00	66.67	100.00
Standard error	0.07	0.09	0.05	0.06	0.07	0.05
Youden Index	0.778	0.778	0.778	0.70	0.67	0.75

AUC, area under curve; Youden's index=sensitivity+specificity-1.

Supplemental Table 4. Top 30 GO annotations for genes targeted by 3 potential miRNAs biomarkers.

GO_ID	Description	Type	*P* value	q value	Enrich factor
hsa-miR-148a-3p					
GO:0002040	sprouting angiogenesis	biological_process	2.49E-06	1.69E-04	6.67
GO:0016441	posttranscriptional gene silencing	biological_process	2.96E-06	1.89E-04	7.44
GO:0035194	posttranscriptional gene silencing by RNA	biological_process	2.96E-06	1.89E-04	7.44
GO:0008038	neuron recognition	biological_process	1.59E-06	1.25E-04	9.51
GO:0035195	gene silencing by miRNA	biological_process	1.09E-05	5.06E-04	7.24
GO:0021795	cerebral cortex cell migration	biological_process	1.79E-05	7.54E-04	6.76
GO:0002042	cell migration involved in sprouting angiogenesis	biological_process	1.37E-05	6.15E-04	8.32
GO:0040036	regulation of fibroblast growth factor receptor signaling pathway	biological_process	4.25E-05	1.51E-03	8.45
GO:0060612	adipose tissue development	biological_process	9.11E-05	2.84E-03	7.36
GO:0090207	regulation of triglyceride metabolic process	biological_process	1.09E-04	3.24E-03	7.13
GO:0016442	RISC complex	cellular_component	5.50E-06	3.07E-04	17.28
GO:0070884	regulation of calcineurin-NFAT signaling cascade	biological_process	6.59E-05	2.16E-03	10.01
GO:0032878	regulation of establishment or maintenance of cell polarity	biological_process	1.05E-04	3.18E-03	9.05
GO:0033173	calcineurin-NFAT signaling cascade	biological_process	3.46E-04	7.73E-03	7.04
GO:0021799	cerebral cortex radially oriented cell migration	biological_process	5.70E-04	1.14E-02	6.34
GO:0035068	micro-ribonucleoprotein complex	cellular_component	1.51E-05	6.63E-04	21.73
GO:0035278	negative regulation of translation involved in gene silencing by miRNA	biological_process	3.65E-05	1.34E-03	16.9
GO:0048268	clathrin coat assembly	biological_process	1.06E-04	3.17E-03	12.67
GO:0031061	negative regulation of histone methylation	biological_process	2.48E-04	6.32E-03	10.14
GO:0021542	dentate gyrus development	biological_process	3.19E-04	7.48E-03	9.51
GO:0007413	axonal fasciculation	biological_process	6.25E-04	1.24E-02	8
GO:0005942	phosphatidylinositol 3-kinase complex	cellular_component	6.25E-04	1.24E-02	8
GO:0005545	1-phosphatidylinositol binding	molecular_function	7.65E-04	1.42E-02	7.6
GO:0031290	retinal ganglion cell axon guidance	biological_process	7.65E-04	1.42E-02	7.6
GO:2000209	regulation of anoikis	biological_process	7.65E-04	1.42E-02	7.6
GO:0048103	somatic stem cell division	biological_process	1.11E-03	1.73E-02	6.91
GO:0035196	production of miRNAs involved in gene silencing by miRNA	biological_process	1.11E-03	1.73E-02	6.91
GO:0043567	regulation of insulin-like growth factor	biological_process	1.57E-03	2.13E-02	6.34

	receptor signaling pathway				
GO:0043276	anoikis	biological_process	1.57E-03	2.13E-02	6.34
GO:0060216	definitive hemopoiesis	biological_process	1.57E-03	2.13E-02	6.34
hsa-miR-362-5p					
GO:0007155	cell adhesion	biological_process	9.38E-04	9.56E-03	3.16
GO:0048514	blood vessel morphogenesis	biological_process	2.31E-04	6.20E-03	4.98
GO:0001568	blood vessel development	biological_process	5.31E-04	7.28E-03	4.39
GO:0001944	vasculature development	biological_process	6.85E-04	8.21E-03	4.22
GO:0016337	single organismal cell-cell adhesion	biological_process	1.46E-04	5.36E-03	6.19
GO:0030155	regulation of cell adhesion	biological_process	2.26E-04	6.16E-03	5.76
GO:0004674	protein serine/threonine kinase activity	molecular_function	4.10E-03	2.03E-02	3.94
GO:0010256	endomembrane system organization	biological_process	4.73E-03	2.24E-02	3.82
GO:0043547	positive regulation of GTPase activity	biological_process	6.33E-03	2.58E-02	3.6
GO:0045664	regulation of neuron differentiation	biological_process	7.05E-03	2.75E-02	3.52
GO:0043087	regulation of GTPase activity	biological_process	1.06E-02	3.52E-02	3.23
GO:0030036	actin cytoskeleton organization	biological_process	1.10E-02	3.61E-02	3.21
GO:0009143	nucleoside triphosphate catabolic process	biological_process	1.16E-02	3.75E-02	3.17
GO:0046130	purine ribonucleoside catabolic process	biological_process	1.19E-02	3.83E-02	3.15
GO:0021510	spinal cord development	biological_process	2.90E-05	1.10E-02	13.97
GO:0021953	central nervous system neuron differentiation	biological_process	3.43E-04	6.11E-03	8.13
GO:0060537	muscle tissue development	biological_process	1.05E-02	3.51E-02	3.69
GO:0048589	developmental growth	biological_process	1.09E-02	3.61E-02	3.66
GO:0043025	neuronal cell body	cellular_component	1.14E-02	3.73E-02	3.62
GO:0032869	cellular response to insulin stimulus	biological_process	1.25E-02	3.92E-02	3.54
GO:0030900	forebrain development	biological_process	1.36E-02	4.15E-02	3.47
GO:0048011	neurotrophin TRK receptor signaling pathway	biological_process	1.38E-02	4.21E-02	3.45
GO:0051493	regulation of cytoskeleton organization	biological_process	1.41E-02	4.27E-02	3.43
GO:0038179	neurotrophin signaling pathway	biological_process	1.43E-02	4.29E-02	3.42
GO:0030425	dendrite	cellular_component	1.47E-02	4.39E-02	3.4
GO:0001525	angiogenesis	biological_process	1.48E-02	4.42E-02	3.39
GO:0071900	regulation of protein serine/threonine kinase activity	biological_process	1.81E-02	5.02E-02	3.22
GO:0032868	response to insulin	biological_process	1.92E-02	5.24E-02	3.18
GO:0044297	cell body	cellular_component	1.92E-02	5.24E-02	3.18
GO:0072657	protein localization to membrane	biological_process	1.93E-02	5.26E-02	3.17
hsa-miR-194-5p					
GO:0007030	Golgi organization	biological_process	4.93E-07	3.19E-04	8.7
GO:0001838	embryonic epithelial tube formation	biological_process	8.57E-06	1.75E-03	6.19
GO:0072175	epithelial tube formation	biological_process	9.16E-06	1.78E-03	6.14
GO:0035148	tube formation	biological_process	2.18E-05	2.22E-03	5.53
GO:0001843	neural tube closure	biological_process	3.68E-06	1.02E-03	7.91
GO:0001841	neural tube formation	biological_process	1.42E-05	2.12E-03	6.62

GO:0033116	endoplasmic reticulum-Golgi intermediate compartment membrane	cellular_component	1.85E-05	2.18E-03	9.06
GO:0017053	transcriptional repressor complex	cellular_component	6.30E-05	3.94E-03	7.37
GO:0006476	protein deacetylation	biological_process	1.02E-04	5.52E-03	6.79
GO:0001948	glycoprotein binding	molecular_function	4.25E-04	1.17E-02	5.32
GO:0005793	endoplasmic reticulum-Golgi intermediate compartment	cellular_component	4.51E-04	1.22E-02	5.27
GO:0003158	endothelium development	biological_process	4.78E-04	1.21E-02	5.21
GO:0048066	developmental pigmentation	biological_process	6.65E-05	4.10E-03	9.15
GO:0016575	histone deacetylation	biological_process	3.48E-04	1.06E-02	6.62
GO:0051149	positive regulation of muscle cell differentiation	biological_process	3.76E-04	1.09E-02	6.52
GO:0070603	SWI/SNF superfamily-type complex	cellular_component	6.25E-04	1.44E-02	5.89
GO:1901800	positive regulation of proteasomal protein catabolic process	biological_process	9.27E-04	1.44E-02	5.44
GO:0046915	transition metal ion transmembrane transporter activity	molecular_function	2.13E-04	8.09E-03	9.56
GO:0060563	neuroepithelial cell differentiation	biological_process	3.00E-04	9.93E-03	8.82
GO:0046928	regulation of neurotransmitter secretion	biological_process	6.06E-04	1.43E-02	7.48
GO:0060425	lung morphogenesis	biological_process	9.40E-04	1.45E-02	6.75
GO:0021879	forebrain neuron differentiation	biological_process	1.29E-03	1.73E-02	6.26
GO:0051219	phosphoprotein binding	molecular_function	1.62E-03	1.71E-02	5.93
GO:0000118	histone deacetylase complex	cellular_component	1.74E-03	1.81E-02	5.83
GO:1901888	regulation of cell junction assembly	biological_process	1.87E-03	1.90E-02	5.74
GO:0032922	circadian regulation of gene expression	biological_process	1.87E-03	1.90E-02	5.74
GO:0021872	forebrain generation of neurons	biological_process	2.29E-03	2.21E-02	5.46
GO:0032436	positive regulation of proteasomal ubiquitin-dependent protein catabolic process	biological_process	2.61E-03	2.14E-02	5.29
GO:0048041	focal adhesion assembly	biological_process	2.78E-03	2.26E-02	5.21
GO:0019935	cyclic-nucleotide-mediated signaling	biological_process	2.78E-03	2.26E-02	5.21

Supplemental Table 5. Top 30 KEGG pathways for genes targeted by 3 potential miRNAs biomarkers.

Pathway ID	Description	*P* value	q value	Count	Enrich factor
hsa-miR-148a-3p					
hsa04510	Focal adhesion	1.08E-05	2.32E-04	18	3.42
hsa04810	Regulation of actin cytoskeleton	8.53E-05	1.00E-03	17	3.02
hsa05215	Prostate cancer	6.15E-07	7.94E-05	13	5.55
hsa04722	Neurotrophin signaling pathway	4.98E-04	4.29E-03	11	3.29
hsa05221	Acute myeloid leukemia	1.78E-06	1.15E-04	10	6.56
hsa05214	Glioma	5.21E-06	2.24E-04	10	5.85
hsa05211	Renal cell carcinoma	1.04E-05	2.68E-04	10	5.43
hsa05410	Hypertrophic cardiomyopathy (HCM)	4.86E-05	6.97E-04	10	4.58
hsa05220	Chronic myeloid leukemia	8.40E-05	1.08E-03	9	4.69
hsa04012	ErbB signaling pathway	3.43E-04	3.69E-03	9	3.93
hsa05414	Dilated cardiomyopathy	4.47E-04	4.12E-03	9	3.8
hsa05213	Endometrial cancer	3.35E-05	6.18E-04	8	5.85
hsa05223	Non-small cell lung cancer	4.48E-05	7.23E-04	8	5.63
hsa05218	Melanoma	3.47E-04	3.44E-03	8	4.28
hsa04350	TGF-beta signaling pathway	1.25E-03	1.01E-02	8	3.58
hsa04512	ECM-receptor interaction	1.25E-03	1.01E-02	8	3.58
hsa04916	Melanogenesis	4.00E-03	1.99E-02	8	3.01
hsa04115	p53 signaling pathway	1.37E-03	9.29E-03	7	3.86
hsa05412	Arrhythmogenic right ventricular cardiomyopathy (ARVC)	2.12E-03	1.25E-02	7	3.6
hsa04662	B cell receptor signaling pathway	2.31E-03	1.30E-02	7	3.55
hsa04070	Phosphatidylinositol signaling system	2.94E-03	1.52E-02	7	3.41
hsa05222	Small cell lung cancer	4.96E-03	2.37E-02	7	3.13
hsa00562	Inositol phosphate metabolism	2.08E-03	1.28E-02	6	4
hsa05212	Pancreatic cancer	6.46E-03	2.87E-02	6	3.26
hsa04150	mTOR signaling pathway	6.23E-03	2.87E-02	5	3.66
hsa05210	Colorectal cancer	1.43E-02	4.98E-02	5	3.07
hsa04320	Dorso-ventral axis formation	1.28E-03	9.20E-03	4	6.08
hsa05216	Thyroid cancer	2.46E-03	1.32E-02	4	5.24
hsa05219	Bladder cancer	1.18E-02	4.34E-02	4	3.62
hsa04710	Circadian rhythm	7.26E-03	2.93E-02	3	4.96
hsa-miR-362-5p					
hsa04510	Focal adhesion	7.37E-03	4.08E-02	3	5.53
hsa04810	Regulation of actin cytoskeleton	9.31E-03	4.47E-02	3	5.16
hsa04960	Aldosterone-regulated sodium reabsorption	6.31E-04	4.54E-02	2	17.54
hsa05223	Non-small cell lung cancer	1.30E-03	4.68E-02	2	13.64
hsa00562	Inositol phosphate metabolism	1.52E-03	3.64E-02	2	12.93
hsa05214	Glioma	2.21E-03	3.97E-02	2	11.33
hsa04971	Gastric acid secretion	3.18E-03	4.59E-02	2	9.96
hsa04370	VEGF signaling pathway	3.43E-03	4.12E-02	2	9.69

hsa04070	Phosphatidylinositol signaling system	3.69E-03	3.80E-02	2	9.45
hsa04664	Fc epsilon RI signaling pathway	3.83E-03	3.44E-02	2	9.33
hsa03008	Ribosome biogenesis in eukaryotes	4.10E-03	3.28E-02	2	9.1
hsa03015	mRNA surveillance pathway	4.39E-03	3.16E-02	2	8.88
hsa04012	ErbB signaling pathway	5.01E-03	3.28E-02	2	8.47
hsa04666	Fc gamma R-mediated phagocytosis	6.40E-03	3.84E-02	2	7.76
hsa05146	Amoebiasis	8.65E-03	4.45E-02	2	6.95
hsa04270	Vascular smooth muscle contraction	1.11E-02	4.97E-02	2	6.35
hsa04670	Leukocyte transendothelial migration	1.13E-02	4.79E-02	2	6.3
hsa-miR-194-5p					
hsa04510	Focal adhesion	1.65E-03	6.71E-02	9	3.27
hsa04010	MAPK signaling pathway	1.25E-02	2.18E-01	9	2.44
hsa04120	Ubiquitin mediated proteolysis	4.81E-04	5.87E-02	8	4.19
hsa04360	Axon guidance	1.41E-03	8.58E-02	7	3.92
hsa04350	TGF-beta signaling pathway	3.12E-03	9.52E-02	5	4.28
hsa04114	Oocyte meiosis	1.27E-02	1.94E-01	5	3.19
hsa04722	Neurotrophin signaling pathway	2.08E-02	2.31E-01	5	2.86
hsa04530	Tight junction	2.55E-02	2.23E-01	5	2.74
hsa04514	Cell adhesion molecules (CAMs)	2.82E-02	2.15E-01	5	2.68
hsa04910	Insulin signaling pathway	3.00E-02	2.03E-01	5	2.64
hsa04720	Long-term potentiation	7.07E-03	1.72E-01	4	4.16
hsa05412	Arrhythmogenic right ventricular cardiomyopathy (ARVC)	8.90E-03	1.81E-01	4	3.93
hsa04512	ECM-receptor interaction	1.56E-02	2.12E-01	4	3.42
hsa04012	ErbB signaling pathway	1.71E-02	2.09E-01	4	3.35
hsa04666	Fc gamma R-mediated phagocytosis	2.42E-02	2.46E-01	4	3.06
hsa04916	Melanogenesis	3.07E-02	1.97E-01	4	2.88
hsa05131	Shigellosis	2.44E-02	2.29E-01	3	3.52
hsa05214	Glioma	2.85E-02	2.05E-01	3	3.36
hsa05211	Renal cell carcinoma	3.63E-02	2.21E-01	3	3.12
hsa05218	Melanoma	3.79E-02	2.20E-01	3	3.07
hsa04520	Adherens junction	4.15E-02	2.30E-01	3	2.99
hsa04662	B cell receptor signaling pathway	4.52E-02	2.21E-01	3	2.91
hsa04370	VEGF signaling pathway	4.71E-02	2.21E-01	3	2.87
hsa05410	Hypertrophic cardiomyopathy (HCM)	6.20E-02	2.61E-01	3	2.63
hsa05219	Bladder cancer	4.42E-02	2.24E-01	2	3.47
hsa04930	Type II diabetes mellitus	6.19E-02	2.70E-01	2	3.03
hsa05144	Malaria	7.20E-02	2.93E-01	2	2.85
hsa04150	mTOR signaling pathway	7.55E-02	2.97E-01	2	2.8
hsa04340	Hedgehog signaling pathway	9.05E-02	3.35E-01	2	2.6
hsa04621	NOD-like receptor signaling pathway	1.03E-01	3.69E-01	2	2.47

Supplementary Table 6. The intersection between 3 potential miRNAs biomarkers and angiogenesis pathway genes.

Systematic Name	The intersected angiogenesis pathway genes	Number
hsa-miR-148a-3p	MEOX2, ROBO1, COL4A1, NRP1, PNPLA6, ACVR1, STARD13, C1GALT1, E2F7, DICER1, F3, S1PR1, TEK, TGFB2, ITGA5, NRARP, KLF5, EPAS1, ROCK1, KLF4, EFNB2, SERPINE1, PTEN, PIK3CA, DDAH1, SULF1, VASH2, NR2E1, PTPRM, NAA15, MMP19, EGR3	32
hsa-miR-362-5p	CYP1B1, DLL1, PRKCA, PIK3CB	4
hsa-miR-194-5p	SLC12A6, PTGIS, ARHGAP24, NRP1, EFNB2, THBS1	6

Supplemental Materials

Supplemental Figure 1 Signal distribution of miRNA microarray of blood samples. The middle line of the figure represents the median of the overall data. The yellow box contains 50% of the overall data, and this area is the Inter Quantile Range.

Supplemental Figure 2 Heat map analysis of 12 differentially expressed miRNAs in PA-HSOS patients in cohort 1 by microarray.

Supplemental Figure 3 Representative UPLC-DAD-MS chromatograms of *G. japonica* total alkaloid extracts. DAD data were acquired from 200-400 nm while MS data were acquired from m/z 50-650.

Supplemental Figure 4 Blood levels of reported miRNAs candidates.

Supplemental Figure 1

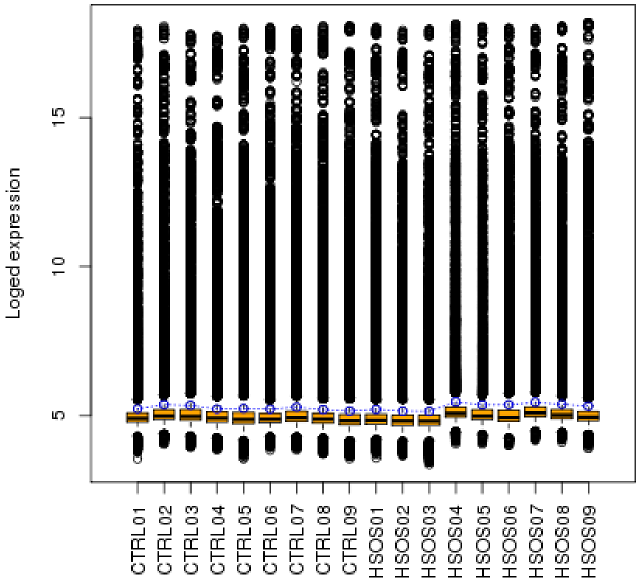

Supplemental Figure 2

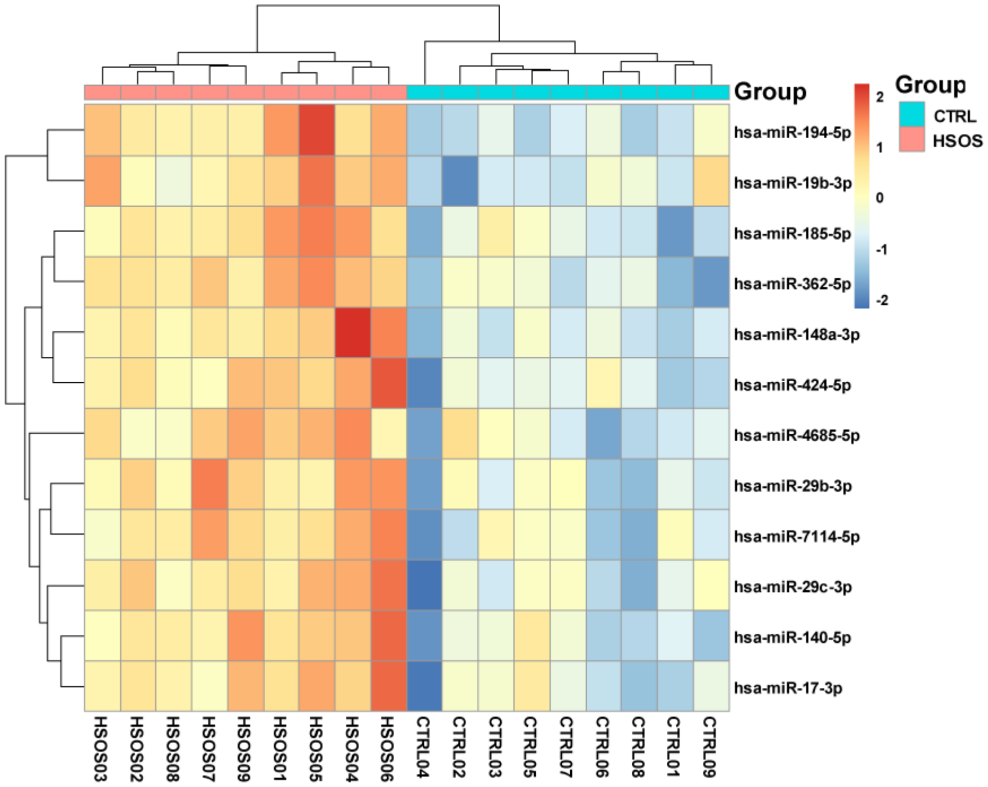

Supplemental Figure 3

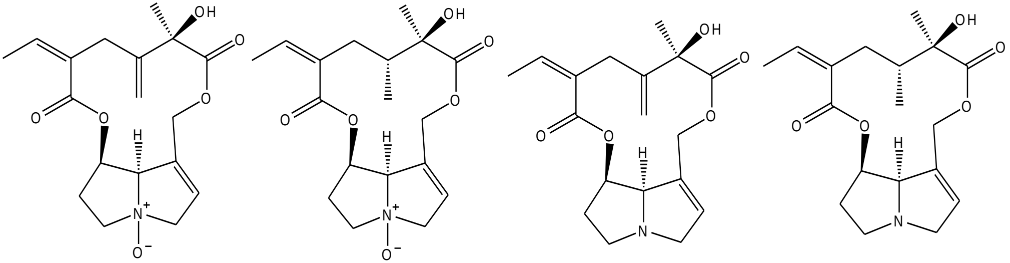

1 seneciphylline *N*-oxide　2 senecionine *N*-oxide　3 seneciphylline　4 senecionine

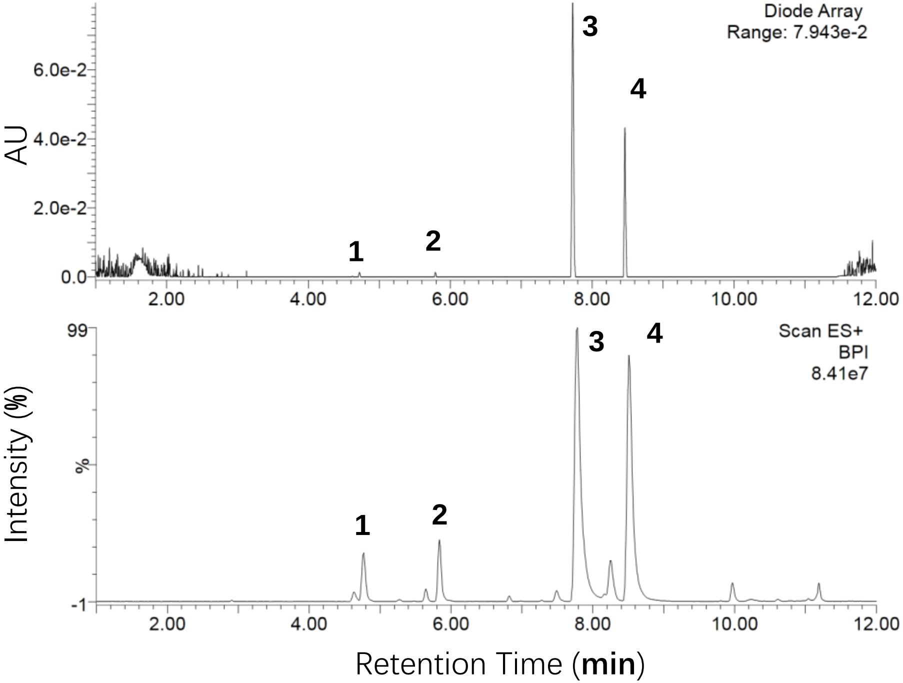

Supplemental Figure 4

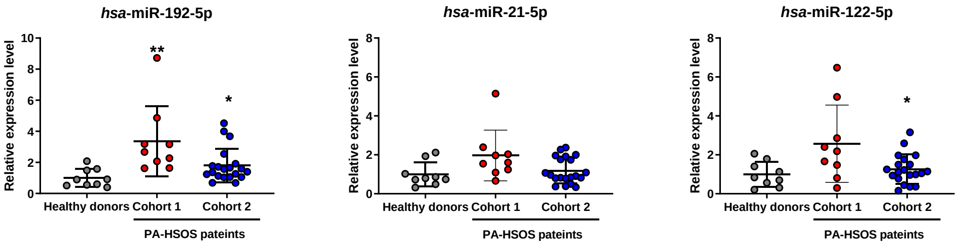

Supplement: Supplementary file 1 [file datasheet1.pdf]
